# Inmates' Experiences of Rehabilitation and Self‐Perception: A Sense of Coherence Exploration From Leeuwkop Correctional Centre

**DOI:** 10.1002/cbm.70045

**Published:** 2026-07-21

**Authors:** Bonginkosi Ndimande, Elizabeth Archer

**Affiliations:** ^1^ Department of Educational Psychology University of the Western Cape Bellville South Africa

**Keywords:** identity transformation, meaning‐making, rehabilitation, repeat offenders, self‐perception, sense of coherence

## Abstract

**Background:**

Rehabilitation is a central objective of correctional services in South Africa, intended to support behavioural change, reduce recidivism and contribute to successful reintegration. Despite this mandate, concerns regarding persistent recidivism and prison overcrowding remain. Less is known about how incarcerated individuals themselves experience rehabilitation and make sense of it.

**Aim:**

To explore how incarcerated individuals describe and make sense of their experiences of rehabilitation within a South African correctional setting.

**Methods:**

Forty repeat‐offending men serving custodial sentences at Leeuwkop Correctional Centre, South Africa, and having taken part in some rehabilitation programmes there, agreed to participate in semi‐structured interviews about their experiences of the rehabilitation work. Data were analysed using inductive thematic analysis. Findings were partially validated through interviews with eight professional staff members who had been delivering aspects of the rehabilitation work. Themes were subsequently interpreted using the Sense of Coherence framework.

**Results:**

Prisoner participants described shifts in self‐perception, including increased self‐awareness, a greater sense of personal responsibility and more future‐oriented perspectives. These experiences broadly fit with the Sense of Coherence components of comprehensibility, manageability and meaningfulness. Such shifts were not, however, uniform and were often described as tentative and shaped by contextual constraints.

**Conclusion:**

Rehabilitation was experienced not only as a behavioural intervention but also as a process of meaning‐making and identity reconstruction. By applying the Sense of Coherence framework as a postanalytic interpretive lens, this study offers a novel way of understanding how incarcerated individuals experience rehabilitation and perhaps internalise key aspects of it. Further longitudinal research is needed to examine the sustainability of these perceived changes over time.

## 1 Introduction

South Africa's correctional system operates within a complex sociohistorical context shaped by high crime rates, deep socioeconomic inequality and the enduring effects of apartheid‐era marginalisation. These structural conditions contribute to persistent challenges within the criminal justice system, including overcrowding, limited rehabilitation resources and a continued emphasis on risk management and behavioural compliance.

Rehabilitation policy, as articulated in the White Paper on Corrections in South Africa (2005), prioritises reducing recidivism and supporting offender reintegration. However, in practice, programme design often remains rooted in control‐oriented and deficit‐based approaches that emphasise risk reduction over personal development (Muntingh 2005). As a result, limited attention is given to how inmates understand themselves and their capacity for change, an issue central to long‐term desistance.

Despite growing recognition of the importance of rehabilitation (Andrews and Bonta 2010), there is limited empirical research examining how rehabilitation programmes influence inmates' self‐perception, particularly in relation to how individuals make sense of their experiences, develop a sense of control and find meaning in their lives during incarceration (Ward 2021).

One framework that may offer insight into these processes is the Sense of Coherence (SOC), which refers to the extent to which individuals perceive their lives as understandable (comprehensible), manageable and meaningful (Vinje et al. 2022). In practical terms, this can be understood as: Do I understand what is happening in my life? Do I have the resources to deal with it? Does it matter enough for me to act? Although Sense of Coherence has been widely applied in health research, its application within correctional rehabilitation remains limited (Antonovsky 1987).

### 1.1 Identity and Self‐Perception in Correctional Contexts

In correctional environments, identity is often shaped by institutional labelling, where individuals are defined primarily as ‘offenders’ or ‘criminals’. Such labels can become internalised, reinforcing stigma, social exclusion and cycles of reoffending (LeBel et al. 2008; Maruna 2012). As a result, how inmates perceive themselves becomes central to understanding behaviour, motivation and pathways to desistance.

Identity in this context is shaped through both social labelling and personal meaning‐making processes. Engagement in structured and prosocial activities, such as education, therapy and restorative practices, has been associated with more positive self‐perceptions (Fulham et al. 2023; South African Department of Justice and Constitutional Development 2021). However, the processes through which such shifts occur remain insufficiently theorised. This highlights the need for conceptual frameworks to help understand how incarcerated individuals make sense of rehabilitation experiences and whether these experiences are associated with changes in self‐perception and identity.

### 1.2 Rehabilitation Approaches and Their Limitations

Contemporary rehabilitation in South Africa, guided by the White Paper on Corrections in South Africa (2005), includes interventions such as cognitive‐behavioural therapy, vocational training and educational programmes. These aim to reduce recidivism and support (re)integration by addressing criminogenic needs and developing practical skills.

The Risk–Need–Responsivity Model remains influential, focusing on risk assessment and targeted intervention (Andrews and Bonta 2010). Although empirically supported, it has been criticised for emphasising risk management over internal transformation (Ward and Maruna 2021). In contrast, the Good Lives Model foregrounds well‐being and prosocial goal attainment (Ward and Fortune 2013), although institutional resources can constrain its implementation.

Desistance theory further highlights the importance of identity transformation, narrative change and social integration in sustaining long‐term behavioural change (Maruna 2012). However, translating these insights into structured correctional programmes remains challenging, particularly in contexts marked by structural inequality.

Across these approaches, a recurring limitation is the limited attention to the internal psychological processes that underpin sustained change (Ndimande 2024). Although programmes may achieve short‐term behavioural outcomes, deeper identity transformation is less consistently addressed.

### 1.3 The Sense of Coherence Framework

The Sense of Coherence framework, developed by Antonovsky (1987), offers a salutogenic perspective on how individuals maintain resilience in the face of adversity—that is, it offers a focus on promoting health and well‐being, not only limiting disease or, here, antisocial behaviours. Sense of Coherence comprises three interrelated dimensions: comprehensibility (the extent to which life is perceived as structured and understandable), manageability (the perception of having resources to cope) and meaningfulness (the extent to which life is experienced as purposeful) (Lindström and Eriksson 2006).

Rather than focusing on deficits, Sense of Coherence emphasises how individuals interpret their experiences and mobilise resources to navigate challenges. This orientation is particularly relevant in correctional contexts, where individuals often face disrupted life trajectories and social marginalisation.

Although widely applied in health and psychological research (Antonovsky 1996), Sense of Coherence has received limited attention within correctional rehabilitation. Its focus on meaning‐making and internal coherence suggests potential relevance for understanding how inmates reinterpret their experiences during incarceration (Ndimande 2024).

### 1.4 Integrating Sense of Coherence Into Rehabilitation: Identifying the Gap

Despite advances in rehabilitation theory, limited research has examined how internal processes such as meaning‐making, perceived control and purpose relate to changes in inmate self‐perception (Ndimande 2024; Ward 2021). Existing studies tend to prioritise external outcomes, such as recidivism or programme participation, with less attention to the psychological processes that may support sustained change.

There is limited empirical work exploring how comprehensibility, manageability and meaningfulness are reflected in inmates' experiences of rehabilitation (Ndimande 2024; Ward 2021). Addressing this gap may contribute to a more nuanced understanding of how rehabilitation influences identity and supports long‐term desistance.

### 1.5 Aims

In this study, therefore, we examine how inmates experience and make sense of rehabilitation. A qualitative approach was adopted to explore their lived experiences and meaning‐making processes within a specific correctional context.

## 2 Methodology

### 2.1 Ethical Considerations

Ethical approval was obtained from the University of the Western Cape Research Ethics Committee. Permission to conduct the study was granted by the Department of Correctional Services and the management at Leeuwkop Correctional Centre. Participation was voluntary, and informed consent was obtained from all participants. Participants were informed of their right to withdraw at any stage without penalty. Confidentiality was ensured using pseudonyms, and all data were securely stored. Interviews were conducted in private settings to protect participant dignity. Given the population's vulnerability, additional safeguards were implemented. Participants were informed of available support services should any distress arise, and care was taken to minimise potential harm during and after the interview process. No correctional officials were present during interviews to ensure participant confidentiality and reduce potential response bias.

### 2.2 Research Design

A qualitative approach within the constructivist framework (Savin‐Baden and Major 2023; S. J. Tracy 2022) was used. Constructivism asserts that reality is socially constructed and depends on how individuals and groups interpret and ascribe meaning to their experiences within a given context (Savin‐Baden and Major 2023). We aimed to explore how incarcerated individuals describe and make sense of their rehabilitation experiences within a South African correctional setting. A single‐site case study approach was used at Leeuwkop Correctional Centre. Case studies focus on exploring a real‐world phenomenon within its boundaries (Ridder 2017; Yin 2018). This approach enabled an in‐depth exploration of subjective experiences that are not readily accessible through quantitative methods.

### 2.3 Research Site

The Leeuwkop Correctional Centre in Gauteng Province, South Africa, is organised into four management sections—Maximum Security, Medium A, Medium B and Medium C—each reflecting different security classifications and stages of incarceration. All sections experience significant overcrowding, which shapes both living conditions and access to rehabilitation programmes. Maximum Security consists of multiple housing units with high inmate density, in which cells often accommodate large numbers of inmates under strict control. Medium C represents a transitional level from Maximum Security and remains relatively restrictive. Medium B and Medium A generally house inmates with lower security classifications, including those approaching potential release. Medium B and Medium A tend to have greater access to structured rehabilitation and developmental programmes (Department of Correctional Services 2020).

Based on institutional structure, observational insight and participant accounts, inmate populations across sections are substantial and fluctuate due to ongoing admissions, transfers and releases (Department of Correctional Services 2022). This dynamic environment forms an important contextual backdrop for understanding participants' rehabilitation experiences. Rehabilitation was interpreted broadly to include educational, psychological, social work, spiritual and related developmental programmes, rather than focusing on a specific intervention. Based on participant accounts, these included anger management, victim–offender dialogue (VOD), restorative justice programmes, ‘New Beginnings’ programmes, sexual offender treatment programmes, substance abuse programmes and life skills training. These programmes were delivered under broader categories, including psychological services, social work interventions and developmental programmes (Government Printers 1998).

### 2.4 Participants and Sampling

A purposive sampling strategy was employed to recruit participants across the four management sections. The sample comprised 40 inmates (excluding three participants involved in the pilot phase), with 10 participants drawn from each section. Inclusion criteria required that inmates be repeat offenders; be serving sentences of three or more years; have engaged in multiple rehabilitation programmes; and have committed serious offences such as murder, rape, robbery and aggravated assault. The inclusion of repeat offenders enabled participants to reflect not only on their current incarceration but also on previous attempts at reintegration and the challenges they experienced upon release. The professional sample comprised eight rehabilitation staff members (psychologists and social workers only, no lay staff) across all four management sections, with experience ranging from one year and seven months to 19 years. They were included to provide institutional and contextual perspectives and to support triangulation (Guba and Lincoln 2005).

The staff were selected for their direct involvement in rehabilitation programmes and their ability to provide informed insights into inmate development and programme processes. Although the exclusion of nonprofessional staff may be considered a limitation, the selected group was best positioned to address the study's focus.

Participants were identified in collaboration with the Case Management Committee (CMC), a multidisciplinary team responsible for inmate profiling and the development of rehabilitation plans within the correctional centre. The CMC assisted in identifying inmates who met the study's eligibility criteria (e.g., repeat offenders, sentence length and programme participation). Identified individuals were then approached by the researcher, provided with full information about the study and invited to participate voluntarily. Participation was not incentivised, and inmates were informed of their right to decline or withdraw without consequences.

Although this process enabled access to information‐rich participants, it may have introduced sampling bias, as initial identification was facilitated through institutional structures. This was mitigated by the researcher's independent verification of participants' eligibility using biographical data collected during interviews and the consistent application of predefined inclusion criteria. Participation remained entirely voluntary and independent of institutional influence. Table 1 presents biographical data used to verify participant eligibility.

**TABLE 1:** Biographical Characteristics of the 40 male participating prisoners.

Characteristic	Distribution
Age	20–30 years: 1; 30–40 years: 10; 40–50 years: 21; 50–60 years: 6; 60–70 years: 2
Race	Black: 31; White: 5; coloured: 3; other/non–South African: 1
Education level	High school: 30; tertiary: 6; primary: 3; special education: 1
Number of convictions	1: 10, 2: 5, 3: 12, 4: 5, 5: 4, > 5: 4
Previous correctional history	All 40 (100%) participants had at least one prior conviction.
Centre sections represented	Maximum, Medium C, Medium B and Medium A
Rehabilitation exposure	All participants had engaged in two or more programmes, including New Beginnings, Crossroads, anger management, Changing Lanes and spiritual counselling. Other programmes are Alpha, Kairos, sexual offender programme (SOP), restorative justice, life skills, hardline and victim–offender dialogue (VOD).
Inclusion criteria met	All participants met the predefined criteria (repeat offenders; ≥ 3‐year sentences; involvement in multiple rehabilitation programmes; serious offences).

### 2.5 Data Collection

Data were collected over 4 weeks using semi‐structured, in‐depth interviews, supplemented by contextual observations and a reflexive or reflective research journal. The primary researcher, a doctoral candidate with formal training in qualitative research methods, conducted all interviews. This training included experience with interview‐based data collection, probing techniques to elicit depth and clarify meaning and thematic analysis. This informed the design, conduct and interpretation of the study.

An open‐ended interview schedule was used to explore how inmates perceived and experienced rehabilitation. Repeat offenders were able to reflect on both current rehabilitation experiences and prior reintegration attempts. The same core interview framework was used for both inmates and professional participants, with role‐specific probing. For example, inmates were asked to describe their understanding of rehabilitation. In contrast, professionals were asked to reflect on how rehabilitation is communicated to inmates and whether such understanding is evident in practice. This enabled direct comparison of perspectives across participant groups. Triangulation was achieved by systematically comparing inmate accounts with professional perspectives to identify convergence, divergence and contextual explanation.

Each interview lasted approximately 45–60 min and was conducted in a private setting within the correctional facility. Interviews were audio‐recorded with participants' consent and transcribed verbatim. The researcher had no prior relationship with participants and clarified their independent academic role to minimise perceived authority and encourage open engagement. Participants were informed that the study was conducted solely for academic purposes and would not influence their incarceration conditions or parole outcomes. As the researcher held no institutional role and the interviews carried no consequences for participants, deliberate misrepresentation (dissimulation) seemed unlikely. Participants were permitted to communicate in any South African language (barring South African Sign Language). Where necessary, interviews were translated and transcribed into English, with care taken to preserve meaning.

### 2.6 Data Analysis

Data were analysed by the primary researcher using ATLAS.ti as a data management and coding support tool. Coding was researcher‐driven rather than software‐generated. The researcher conducted initial open coding, followed by iterative refinement into higher‐order categories and themes. The software facilitated the organisation, retrieval and comparison of coded data but did not determine the coding framework. Analytical decisions were guided by the researcher's training in qualitative methods and engagement with relevant methodological literature. The analysis followed six stages: familiarisation with the data, line‐by‐line coding, theme development, review and refinement of themes, definition and naming of themes, and final reporting (Savin‐Baden and Major 2023). Both inductive and deductive strategies were employed. Initial coding was inductive, allowing themes to emerge from the data. The Sense of Coherence framework was not used to generate initial codes but was applied during later stages of interpretation, where emergent themes were examined in relation to its core concepts.

Credibility of the analysis was enhanced through structured peer debriefing and ongoing critical reflection. Peer debriefing involved regular discussions between the primary researcher and a co‐author with extensive experience in qualitative research. During these sessions, emerging codes, theme development and interpretive decisions were critically reviewed and refined to ensure that findings remained grounded in the data. These discussions provided a mechanism for challenging assumptions and strengthening analytical rigour. In addition, the primary researcher maintained a reflexive journal to document analytical decisions and reflect on potential biases throughout the research process.

### 2.7 Trustworthiness

Trustworthiness was established using the criteria of credibility, transferability, dependability and confirmability (Lincoln and Guba 1985; A. Tracy 2013). Credibility was established through triangulation, word‐for‐word transcription of data and peer debriefing. Transferability was demonstrated through rich descriptions. Dependability was established through the documentation of the research process and the availability of an audit trail. Confirmability was supported by reflexive journalling, critical analysis and rigorous evaluation of the data coding and the derived themes.

Triangulation involved comparing inmate and professional interview data to achieve cross‐validation and consistency. This process enabled the identification of converging and diverging perspectives and provided contextual insight into institutional practices. Professional accounts were used to contextualise and interpret inmate experiences by explaining programme intentions, processes and constraints, rather than superseding inmate perspectives.

Negative case analysis was conducted throughout the analytical process, with contradictory accounts examined to refine and strengthen interpretations.

## 3 Results

### 3.1 Overview of Findings

Four interrelated themes of participants' experience of rehabilitation emerged from the analysis:

1. Disrupted and stigmatised identity
2. Emerging self‐awareness through reflection
3. Developing agency and personal responsibility
4. Reconstructing purpose and future orientation

These themes reflect recurring patterns across participant accounts while also highlighting important variations in the depth, consistency and sustainability of perceived change.

#### 3.1.1 Disrupted and Stigmatised Identity

Participants frequently described their pre‐rehabilitation self‐perception as fragmented and shaped by negative labelling. Many internalised identities, such as ‘criminal’, ‘failure’ or ‘nothing’, influenced both behaviour and future expectations.

You don’t see yourself as a person anymore, just an offender… that’s what you become inside.

(Med C. Inmate M)

Several participants linked this negative internalisation to repeated incarceration and social rejection: ‘When you come back again and again, you feel like that's who you are… like you can't be anything else’ (Med A. Inmate J). He also reflected on other emotional consequences of incarceration and lost time: ‘Rehabilitation taught me that time is moving, and I have wasted quite some time… I even lost my mother while in prison’.

This sense of disruption was also connected to the loss of autonomy and freedom. Med A. Inmate I explained: ‘Now that I am in prison, I have limited choices… I have lost my freedom, and I need my freedom’.

A professional participant similarly observed that many inmates enter rehabilitation with deeply entrenched negative self‐perceptions shaped by repeated failure, rejection and institutional labelling. Med A. SW4 noted: ‘Many offenders come in already defeated… they don't see themselves as capable of change…. Their identity is already shaped by years of being labelled—by society and by themselves’.

These converging accounts suggest that, before the introduction of rehabilitative programmes, a sense of stigmatised identity is present and may shape how individuals initially respond to programmes.

#### 3.1.2 Emerging Self‐Awareness Through Reflection

Men who had attended rehabilitation programmes, particularly those programmes incorporating dialogue, counselling and structured reflection, reported increased self‐awareness, often for the first time. Participants described reflecting critically on their life histories, decision‐making patterns and the consequences of their actions.

Several participants described rehabilitation as exposing them to perspectives and forms of self‐understanding they had not previously considered. ‘The programmes made me think about my life properly for the first time… why I made certain choices’. Med A. Inmate E. Similarly, Med B. Inmate LE reflected: ‘I got to understand why and what triggered the act that got me arrested. You also discover why you went through the wrong way’. ‘I had to look at myself honestly… not blame others but really see where I went wrong’. Med C. Inmate V. Some participants described this process as uncomfortable but necessary: It’s not easy… you have to face things you avoided for years (Med B. Inmate Y.).

Staff members emphasised reflection as a key mechanism of change: ‘When offenders start reflecting, that's when you see a shift… they begin to take ownership’ (Max. SW2). ‘Programmes that just give information don't work the same—reflection is what makes it real’ (Med C. P1).

Some participants seemed aware of a distinction between attendance and engagement. A minority described a limited perceived value: ‘Some people just attend because they must… it doesn't really change how they think’ (Max. Inmate LK). This variation was reinforced by Med B. Inmate Y, who stated: ‘Rehabilitation starts from within. You must want to do it’. This variation suggests that reflective engagement is a critical, but not guaranteed, component of rehabilitation.

#### 3.1.3 Developing Agency and Personal Responsibility

A central theme across participant accounts was a shift from externalising blame to recognising personal responsibility. Participants described moving away from narratives focused on circumstances or others towards greater acknowledgement of their own role:

‘Before, I blamed everyone… now I see I had a part to play’ Med A. Inmate C. Participants frequently linked this shift to increased emotional regulation and behavioural control. Med A. Inmate C explained: ‘The anger management programme has helped me to control my anger… all these programmes have changed the way I have been and now I have goals’.

A growing sense of agency and perceived control often accompanied this shift. Med B. Inmate Y reflected: ‘Before, if somebody attacked me verbally or physically, I would retaliate. But now I have outgrown that’. Max. Inmate LL described a changed understanding of conflict: ‘The guy that wins the fight is the one that turns away and walks away’. These narratives suggest that participants increasingly interpreted restraint and self‐regulation as indicators of personal strength rather than weakness.

Staff participants corroborated these observations: ‘You can see when someone starts taking responsibility—it shows in their behaviour and decisions’ (Med B. P3). ‘The change is when they stop seeing themselves as victims of circumstances’ (Med B. SW1).

At the same time, some participants expressed uncertainty about sustaining this agency outside the structured prison environment. Max. Inmate LQ noted, ‘Here it's easier to control things… outside it might be different’. This uncertainty suggests that although agency may develop internally, its application remains context‐dependent.

#### 3.1.4 Reconstructing Purpose and Future Orientation

Many participants described a shift towards future‐oriented thinking and the development of more positive, prosocial identities. These included aspirations related to family, employment and social contribution. Similarly, Med C. Inmate M reflected on his desire to rebuild relationships and redefine himself beyond his criminal past: ‘Now I want to be someone my family can be proud of… not go back to the same life’.

Several participants emphasised that their engagement in rehabilitation extended beyond parole or institutional compliance. Max. Inmate LH stated: ‘I do not do this for the certificate; I do it for myself’. This suggests that rehabilitation was, for some participants, experienced as personally meaningful rather than merely procedurally necessary.

Participants also described practical aspirations linked to employment, entrepreneurship and self‐sufficiency. Max. Inmate LA explained: ‘We should not always expect employment… sometimes we must use our hands and be entrepreneurial’. Similarly, Med A. Inmate F stated: ‘I know how to bake, sew, and cook, which are skills I learnt in prison’. These accounts indicate that participants often connected rehabilitation with both personal transformation and practical preparation for reintegration.

Participants often linked this shift to a renewed sense of purpose: ‘Before, I didn't care what happened to me… now I feel like my life has direction’ (Max. Inmate LH). Staff members similarly noted that purpose was associated with sustained engagement: ‘When they start talking about the future differently, you know something has changed’ (Med C Inmate P1).

Some participants, however, expressed concern about structural barriers postrelease: ‘Even if you change, society doesn't always accept you’ (Max. Inmate LC). This suggests recognition of tensions between internal transformation and external realities.

### 3.2 Variation in Experiences

Although positive accounts predominated across participant narratives, experiences of rehabilitation were not uniform. Although most participants described some degree of reflection, learning or perceived personal change, a minority expressed reservations about aspects of programme delivery, relevance or implementation. Some participants appeared to engage with certain programmes primarily for sentence progression or parole‐related purposes. In contrast, others questioned repetitive programme content, the use of inmate‐facilitated courses or the lack of interventions tailored to individual needs. These negative cases illustrate that the perceived value and personal significance of rehabilitation varied across participants and programme contexts.

## 4 Discussion

Our findings indicate that most participants experienced at least some personally meaningful inner change as they progressed through the elements of rehabilitation, including a shift from disrupted and stigmatised identities towards greater self‐awareness, personal responsibility and future orientation. These shifts were neither uniform nor linear, but they suggest internal processes that are likely to be important and subject to change through effective rehabilitation.

These accounts are participants' perceptions and interpretations of change rather than objective measures of transformation. Further, we could not measure any actual changes over time. Rather, we captured participants' retrospective and current interpretations of their experiences of imprisonment at a single point in time. Nevertheless, inner thoughts and feelings can only be described by the people experiencing them, and they volunteered these ideas of change; no questions directed or led them in this direction.

A central contribution of this study is the use of the Sense of Coherence framework as an interpretive lens to organise and deepen understanding of these accounts. The framework was applied after themes were developed inductively, enabling a theoretically informed interpretation without constraining the initial analysis.

### 4.1 Reinterpreting Identity Through Comprehensibility

Participants' accounts of reflection and increased self‐awareness suggest a developing capacity to make sense of their past actions and current circumstances. Having described their preprison and early lives as disrupted and stigmatised and often experienced as chaotic or shaped by external circumstances, many reported becoming more self‐aware. They described a greater sense of personal influence over their future choices.

This interpretive shift aligns with what Antonovsky (1987) conceptualised as comprehensibility, the extent to which individuals perceive life events as ordered and understandable. Within desistance research, similar processes have been described as ‘cognitive transformation’ or ‘narrative reconstruction’, where individuals reframe their past in ways that support change (Maruna 2012).

However, the present findings suggest that such meaning‐making is not automatic. Participants varied in their depth of reflection, with some engaging superficially or primarily for instrumental reasons (e.g., parole progression). This indicates that opportunities for reflection alone may be insufficient; in practice, checks of active engagement and readiness for change may be essential for identifying those who need additional help to achieve change.

### 4.2 Interpreting Agency Through Manageability

Participants frequently described increased self‐control, responsibility and confidence in managing challenges. These accounts suggest an emerging sense of personal agency, which may be interpreted through Antonovsky's (1987) concept of manageability. Rather than suggesting complete mastery or control, participants frequently described an emerging sense of capability and behavioural regulation within the constraints of incarceration. Participants described acquiring cognitive and emotional tools that enabled them to respond differently to challenges, suggesting a shift from externalising blame to recognising agency (Ward 2021).

This finding extends existing critiques of the Risk–Need–Responsivity Model (Ward and Fortune 2013) by highlighting that addressing the concept of ‘criminogenic needs’ may not fully capture how individuals come to see themselves as capable of managing their lives. In this study, participants' narratives suggest that perceived capability (rather than skill acquisition alone) may be central to the experience of rehabilitation.

Participants frequently acknowledged that life after release would present challenges, including stigma, unemployment and social exclusion. Rather than undermining their emerging sense of agency, these concerns often reflected an awareness of the realities they expected to face upon reintegration. The findings suggest that rehabilitation may support the development of personal responsibility and self‐regulation within the correctional environment, while also highlighting the importance of helping individuals consider how these capacities can be applied and sustained in different social and community contexts.

### 4.3 Reconstructing Purpose Through Meaningfulness

Participants' descriptions of developing goals and aspirations, and of a desire to ‘be someone different’, reflect elements of meaningfulness. Many articulated a renewed sense of purpose linked to family, social contribution or personal growth.

This resonates with the Good Lives Model (Fortune and Ward 2013; Viglione 2019), which emphasises the role of meaningful goals in sustaining desistance. However, the present findings suggest that meaningfulness is experienced subjectively and unevenly. Although some participants articulated clear future‐oriented identities, others expressed uncertainty or ambivalence.

These variations highlight that the development of purpose is not simply an outcome of programme participation but a negotiated process shaped by individual histories, institutional context and perceived future opportunities. It seems important that meaningfulness was not expressed solely through abstract hope but through clearly reported relationships and practical aspirations involving family responsibility, employment, spirituality and social contribution.

### 4.4 Beyond Individual Change: The Role of Context

This finding aligns with broader rehabilitation literature emphasising the importance of social context, including employment, family support and community acceptance, in sustaining desistance (Bushway and Paternoster 2014). This study adds to this by showing how participants themselves anticipate these challenges, often qualifying their own sense of change.

As such, rehabilitation cannot be understood solely as an individual psychological process. Without supportive postrelease conditions, internally constructed identities may be difficult to sustain in practice.

Participants also identified structural and institutional barriers that could undermine rehabilitation efforts, including stigma, unemployment, gang influences, substance abuse and inconsistent programme implementation. These findings suggest that internal shifts in self‐perception may remain vulnerable without sustained structural and relational support beyond incarceration.

### 4.5 Implications for Rehabilitation Practice

The findings suggest that rehabilitation programmes may be strengthened by incorporating elements that support:

- Structured and facilitated self‐reflection
- Development of practical and cognitive coping strategies
- Opportunities to explore and construct meaningful future identities

Rather than replacing existing models, such approaches may complement risk‐based and skills‐based interventions. By incorporating these approaches into interventions and always considering how individuals interpret their work and internalise their experiences of it (Lötter 2019; Ward and Langlands 2009).

Our findings also indicate that perceived programme value depends not only on programme content but also on participant engagement, suggesting a need for fostering and measuring engagement (Higley et al. 2019).

### 4.6 Limitations

This study has limitations. It is based on a single‐site, cross‐sectional design and relies on self‐reported accounts, which may be influenced by recall, social desirability or institutional context. Participants were not assessed at multiple time points; therefore, the study does not measure change over time.

Participants were identified through the Case Management Committee according to the study inclusion criteria. This process may have resulted in greater representation of inmates who were more engaged in rehabilitation programmes, potentially limiting the range of perspectives captured. Although staff interviews were used to contextualise the findings, the analysis primarily reflects inmate narratives. The study also relies on retrospective self‐reporting, which may be shaped by memory, context or perceived expectations within the correctional environment.

### 4.7 Contribution to Knowledge

Rehabilitation programmes are generally evaluated, if they are evaluated at all, according to whether participants are judged to have resolved or managed the problem that brought them into the programme. In health contexts, this is usually assessed through indicators such as relapse or rehospitalisation, whereas in criminal justice contexts, it is assessed through reoffending or desistance. The Sense of Coherence framework can serve as a useful tool for understanding whether and how individuals change during rehabilitation and, with further research, how such changes relate to desistance from offending and healthy community integration.

By linking themes of reflection, agency and purpose to comprehensibility, manageability and meaningfulness, the study offers a way to integrate identity‐based and strengths‐based perspectives in rehabilitation research. The Sense of Coherence framework offers a plausibly useful conceptual language for measuring and interpreting change.

## 5 Conclusion

In open interviews with incarcerated individuals in different wings of the prison, we found prisoners willing to talk about their experiences of rehabilitation and their perceptions of self within a correctional context. Our findings indicate that many, if not most, participants understand rehabilitation not only as required participation in specified time‐tabled events but also as involving shifts in how they see themselves, their experiences and their future. Participants' accounts suggest that engagement in rehabilitation programmes can be associated with increased self‐awareness, a greater sense of personal responsibility and the development of future‐oriented perspectives, although these changes were not uniform. Interpreting them through the Sense of Coherence framework provides a useful way to understand how participants describe making sense of their experiences (comprehensibility), perceiving themselves as capable of responding differently (manageability) and identifying reasons to pursue change (meaningfulness), thereby informing measurable improvements in rehabilitation programmes. Our study adds to the literature on correctional rehabilitation by highlighting the internal, meaning‐making dimensions of change that may be important, even essential, to desistance from further criminal behaviour and positive societal membership.

## Funding

The authors have nothing to report.

## Ethics Statement

Ethical approval was obtained from the University of the Western Cape Research Ethics Committee (HS21/10/63). Permission to conduct the study was granted by the Department of Correctional Services and the management at Leeuwkop Correctional Centre.

## Consent

Informed consent was obtained from all participants prior to data collection, in accordance with ethical research guidelines.

## Conflicts of Interest

The authors declare no conflicts of interest.

## Permission to Reproduce Material From Other Sources

The authors have nothing to report.

## Data Availability

The data that support the findings of this study are available from the corresponding author upon reasonable request.
